# Le schwannome malin du nerf grand sciatique chez l'enfant

**Published:** 2012-08-21

**Authors:** Maryem Lechqar, Imad Elbiache, Karima Atarraf, Youssef Bouabdellah, My Abderahman Afifi

**Affiliations:** 1 Service de Traumatologie Orthopédie Pédiatrique; CHU Hassan II, Fès, Maroc

**Keywords:** Schwannome, malin, nerf grand sciatique, histologie, Schwannoma, Malignant, sciatic nerve, histology

## Abstract

Le schwannome malin est une tumeur très rare chez l'enfant (1 à 2% des tumeurs des tissus mous), elle se développe au dépend des cellules de schwanne. Dans ce travail, les auteurs rapportent un cas de schwannome malin développé au dépend du nerf grand sciatique. La radiographie de la cuisse de face et de profil était normale. L'imagerie par résonance magnétique a identifié une lésion le long du trajet du nerf grand sciatique. La tumeur a été réséquée en totalité emportant le nerf grand sciatique. L'examen anatomo-pathologique a confirmé le diagnostic.

## Introduction

Le schwannome malin est une tumeur est une tumeur très rare chez l'enfant [1], due à la prolifération des cellules de schwanne [2]. Il atteint habituellement les grands troncs nerveux [3, 4]. Il se présente sous la forme d'une masse encapsulée entrainant parfois des déficits neurologiques. La chirurgie se présente comme le seul traitement efficace, la tumeur étant chimio et radio résistante. Le pronostic dépend essentiellement du diagnostic précoce et de la qualité de la résection chirurgicale [1].

## Patient et observation

Il s'agit de Z D, patiente âgée de 14 ans sans ATCD pathologiques particuliers, se présentant en consultation pour une tuméfaction de la face postérieure de la cuisse gauche, évoluant depuis un mois. C’était une masse qui augmentait progressivement de volume, douloureuse et gênant la marche.

**Figure 1:**
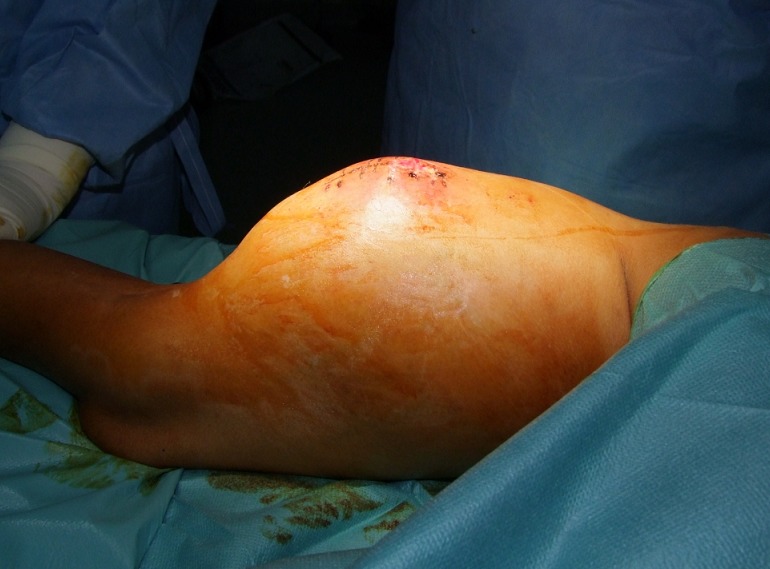
Vue postérieure de la cuisse montrant l’énorme masse

L'examen clinique avait trouvé un enfant apyrétique en ABEG, avec à l'examen loco-moteur une énorme masse de la face postérieure de la cuisse mesurant environ 20cm sur 10 (Figure 1), de consistance dure, losangique, épousant la forme de la cuisse, fixe au deux plans et ne présentant pas de signes inflammatoires en regard.

L'examen neurologique avait objectivé un déficit de la dorsi-flexion du pied avec une mobilité des orteils impossible sans déficit sensitif. L'examen dermatologique a trouvé plusieurs taches café au lait dont le nombre était supérieure à 6 et la taille à 1,5 cm.

**Figure 2:**
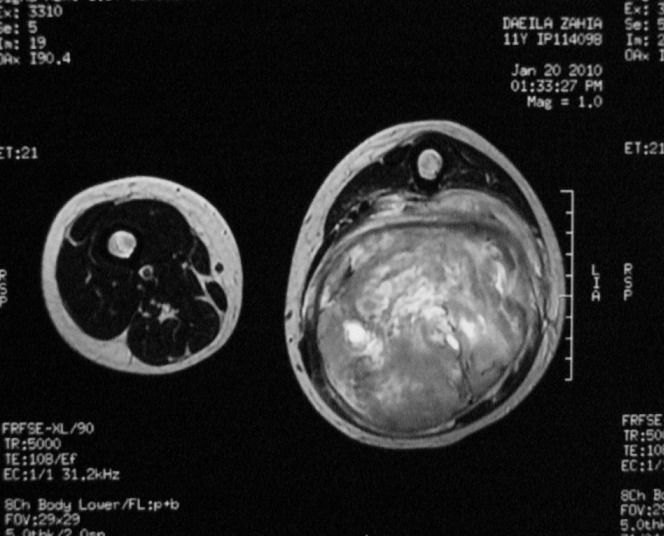
Coupes transversales de la masse en T1 et T2

La radiographie standard du membre était sans particularité, alors que l'IRM était en faveur d'une masse tumorale hémorragique des parties molles postérieures de la cuisse, englobant le muscle biceps crural, le nerf grand sciatique et respectant les autres muscles, le fémur et le pédicule vasculaire (Figure 2).

**Figure 3:**
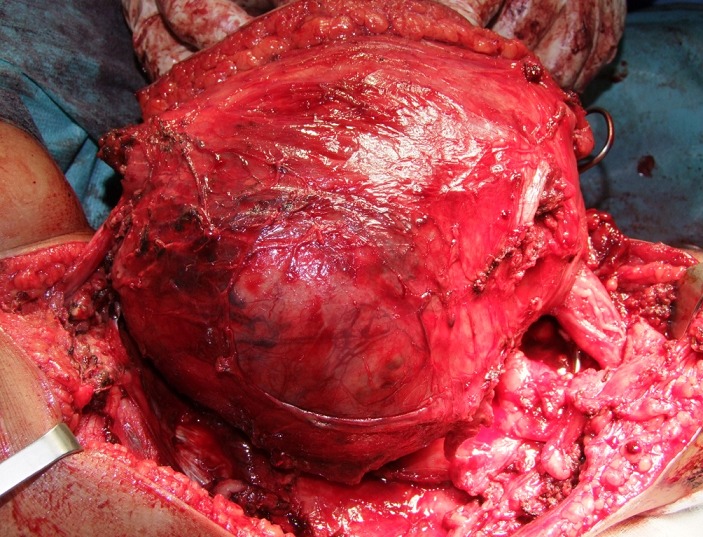
Aspect per-opératoire de la masse englobant le nerf grand sciatique

**Figure 4:**
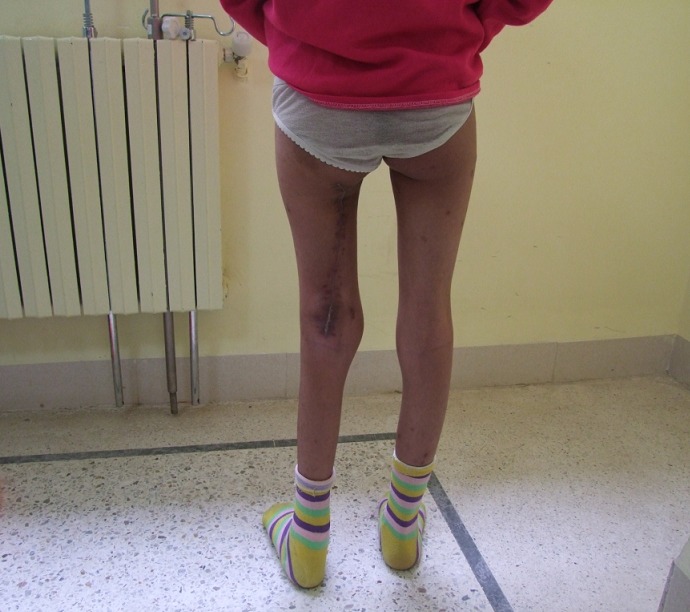
Aspect clinique post opératoire

La malade a bénéficié d'une biopsie, le résultat de l'examen est revenu en faveur d'un sarcome des parties molles, par la suite la patiente a été admise pour résection tumorale. L'exploration avait trouvé une tumeur au dépend du nerf grand sciatique (Figure 3) respectent le pédicule fémorale et l'os. La résection tumorale était complète emportant le nerf grand sciatique alors que examen anatomopathologique de la pièce opératoire a posé le diagnostic d'un schwannome malin avec des limites de résections saines. L’évolution était favorable avec un recul de 6mois (Figure 4). Un an en post-opératoire, l'enfant a présenté une récidive de la tumeur pour laquelle elle a malheureusement eu une désarticulation de la hanche.

## Discussion

Les tumeurs primitives des nerfs périphériques représentent 1 à 2% des tumeurs des tissus mous [1]. Il convient de distinguer les tumeurs malignes survenant généralement dans le cadre de la maladie de Von Reclinghausen des tumeurs nerveuses bénignes essentiellement les schwannomnes bénins et les neurofibromes [5].

Le schwannome malin se développe au dépend des cellules de Schwanne, formant une prolifération macroscopiquement lisse, arrondie et encapsulée. Il atteint habituellement les grands troncs nerveux, en particulier du membre supérieur [2–4]. Au niveau du membre inférieur, les nerfs les plus souvent atteints sont le nerf grand sciatique comme c'est le cas de notre malade et le tibial postérieur au niveau du canal tarsien. Il s'agit de tumeurs très rares chez l'enfant, elle atteint le plus souvent l'adulte entre 25 et 40 ans, ce sont en général des lésions solitaires. Les schwannomes multiples sont rares et doivent faire écarter la possibilité d'une neurofibromatose, leur taille varie entre 4 et 5 cm de diamètre [5]. La tumeur est habituellement bien limitée et encapsulée, et indolore bien qu'il puisse y avoir parfois une inflammation locale douloureuse, des paresthésies des hypoesthésies et rarement une paralysie. L’échographie montre généralement une tumeur pleine et l’étude des rapports nerf-hôte n'est pas facile, l'IRM est l'examen de choix pour l’étude de la tumeur et sa continuité avec le nerf le [6–8], le diagnostic définitif est histologique. Le traitement consiste en l'exérèse chirurgicale complète de la tumeur. Le pronostic de ces tumeurs est variable et le taux de récidive dépend de la résection chirurgicale. Après chirurgie, la survie est de 79% si l'exérèse est complète, 22% si l'exérèse est impossible ou en de métastases [1]. Ce cas présente une énorme tumeur isolée, associée à une neurofibromatose, de localisation au niveau du nerf grand sciatique, les manifestations cliniques étaient la grande masse au niveau de la face postérieure de la cuisse avec la paresthésie du même membre. Le diagnostic a était confirmé histologiquement et l'exérèse a été complète et à limites de résection saines emportant malheureusement le nerf grand sciatique. Les suites opératoires étaient simples.

## Conclusion

Le schwannome malin du nerf grand sciatique est une tumeur nerveuse rare chez l'adulte exceptionnelle chez l'enfant dont le diagnostic est histologique. Le pronostic est bon si l'exérèse chirurgicale est complète. Cependant, un suivi ultérieur est nécessaire pour déceler une éventuelle neurofibromatose.
